# Generation of a virtual cohort of TAVI patients for in silico trials: a statistical shape and machine learning analysis

**DOI:** 10.1007/s11517-024-03215-8

**Published:** 2024-10-10

**Authors:** Roberta Scuoppo, Salvatore Castelbuono, Stefano Cannata, Giovanni Gentile, Valentina Agnese, Diego Bellavia, Caterina Gandolfo, Salvatore Pasta

**Affiliations:** 1 Department of Engineering, Università degli Studi di Palermo, Viale Delle Scienze Ed.8, Palermo, Italy; 2 Department of Research, IRCCS ISMETT, via Tricomi, 5, Palermo, Italy; 3 Interventional Cardiology Unit, IRCCS ISMETT, via Tricomi, 5, Palermo, Italy; 4 Radiology Unit, Department of Diagnostic and Therapeutic Services, IRCCS ISMETT, Via Tricomi, 5, Palermo, Italy

**Keywords:** Transcatheter aortic valve replacement, Statistical shape analysis, Machine learning

## Abstract

**Purpose:**

In silico trials using computational modeling and simulations can complement clinical trials to improve the time-to-market of complex cardiovascular devices in humans. This study aims to investigate the significance of synthetic data in developing in silico trials for assessing the safety and efficacy of cardiovascular devices, focusing on bioprostheses designed for transcatheter aortic valve implantation (TAVI).

**Methods:**

A statistical shape model (SSM) was employed to extract uncorrelated shape features from TAVI patients, enabling the augmentation of the original patient population into a clinically validated synthetic cohort. Machine learning techniques were utilized not only for risk stratification and classification but also for predicting the physiological variability within the original patient population.

**Results:**

By randomly varying the statistical shape modes within a range of ± 2σ, a hundred virtual patients were generated, forming the synthetic cohort. Validation against the original patient population was conducted using morphological measurements. Support vector machine regression, based on selected shape modes (principal component scores), effectively predicted the peak pressure gradient across the stenosis (*R*-squared of 0.551 and RMSE of 11.67 mmHg). Multilayer perceptron neural network accurately predicted the optimal device size for implantation with high sensitivity and specificity (AUC = 0.98).

**Conclusion:**

The study highlights the potential of integrating computational predictions, advanced machine learning techniques, and synthetic data generation to improve predictive accuracy and assess TAVI-related outcomes through in silico trials.

**Graphical Abstract:**

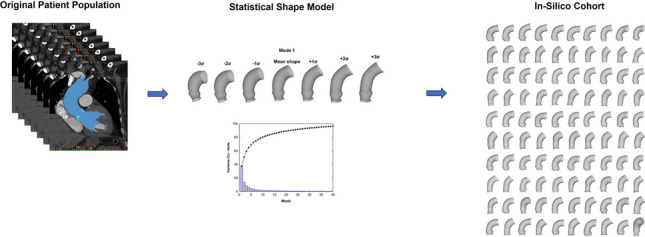

## Introduction

Transcatheter heart valves need many tests in the development stage and relies on clinical trials for demonstrating the safety and efficacy of the intended medical treatment. As in other engineering fields and industries, the design and efficacy of biomedical devices can be greatly improved by using computer modeling and simulations, which can play a pivotal role in accelerating the design phase and thus help companies develop more effective and reliable solutions [1]. Clinical trials offer a detailed assessment and validation of the cardiovascular device in the clinical environment but are costly, time-consuming, and have limited ability to acquire data on outlier patients [2].

There is therefore an emerging interest in developing in silico trials to provide clinically oriented data and improve the time-to-market of complex cardiovascular devices in humans [3]. In silico trials can augment an original patient population to obtain evidences in synthetic models using simulation and provide novel insights in borderline anatomies commonly excluded from clinical trials [4]. For instance, in the setting of transcatheter aortic valve replacement (TAVI), initial clinical trials [5] excluded patients with bicuspid aortic valve or young individuals. Later, specific trials extended the clinical outcome on the safety and efficacy of TAVI in stenotic bicuspid aortic valves [6] with life expectancy longer than the originally treated TAVI patient population. In silico trials can be used to investigate the causes of device underperformance in complex anatomies, which can in turn inform improvements in device design.

To implement an in silico clinical trials for TAVI, a synthetic cohort of patients with different degrees of aortic valve stenosis needs to be developed from an original patient population. Thus, the variability of the targeted patient population should be quantified and expanded to generate a virtual cohort mimicking the patients’ anatomies. To accomplish this task, statistical shape modeling (SSM) represents a powerful technique to extrapolate shape features that, if combined with machine learning models, can also provide predictions on the mechanistic link between shape and function [7]. The virtual cohort should be clearly credible and replicate the morphological and functional characteristics of the original patient population. Thus, the application of in silico trials for providing clinical evidence requires demonstration and the establishment of a regulatory framework [8].

In this study, we propose a framework to extract shape features of TAVI patients using geometric decomposition techniques. As a proof-of-concept, a synthetic cohort of one hundred patient models was derived and then validated against geometric parameters computed for both the synthetic and original TAVI study group. Later, a regression model was developed aimed at directly inferring the pressure gradient of stenotic valve based on the information derived from the previously computed shape features. Correlations between shape modes and functional patient parameters were also quantified. In particular, regression and correlation analyses were conducted to explore the feasibility of estimating functional parameters for synthetic models based on the functional clinical data of the original population and the shape features. As a second aim, we also explored the feasibility of predicting the optimal size of the bioprosthesis from the SSM-related anatomic features using machine learning. This can be potentially used for stratification of borderline anatomies at risk of underperformance of the implanted device.

## Materials and methods

A prospective clinical study was conducted to enroll 68 patients undergoing TAVI with the Edwards SAPIEN 3 (S3) transcatheter heart valve. Patients were recruited within the scope of the H2020 project SimInSitu. They presented varying degrees of aortic valve stenosis and were treated with device sizes of 23 mm or 26 mm. Patients treated with the 29-mm device were not included, as it is rarely used in our hospital institution (incidence < 5%). All patients underwent a rigorous diagnostic imaging protocol. Upon in-hospital admission, both transthoracic echocardiography and electrocardiogram-gated computed tomography (ECG-gated CT) imaging were performed to plan the TAVI procedure. Clinical procedures were performed by the Heart team using transfemoral access under general anesthesia. The implantation depth of the S3 device was established in accordance with the manufacturer’s recommendations and patient anatomic constraints. Table 1 summarizes demographic and clinical data for each patient.

**Table 1:** Study population characteristics of patients prior TAVI procedure

	TAVI patient
	(*N* = 68)
Age (years)	80.52 ± 5.99
Height (cm)	159.25 ± 8.54
mass (kg)	71.17 ± 13.55
BMI (-)	28.11 ± 4.88
BSA (m^2^)	1.77 ± 0.20
P_sys_ (mmHg)	127.15 ± 19.13
P_dia_ (mmHg)	63.27 ± 10.92
Heart rate (bpm)	71.25 ± 9.87
ECG-gated CT data	
Valve area AVA (cm^2^)	0.61 ± 0.13
Indexed valve area (cm^2^)	0.35 ± 0.07
Peak gradient (mmHg)	79.11 ± 14.59
Mean gradient (mmHg)	48.58 ± 9.87
Maximum jet velocity (m/s)	4.40 ± 0.50
EF %	59.82 ± 7.82
PAPs (mmHg)	32.72 ± 11.75
Calcium score (AU)	2120 ± 770
Stroke volume (ml) Stroke volume/BSA (ml/m^2^)	58.51 ± 14.95
Cardiac output (l/min)	4.11 ± 1.15
End diastolic left ventricular volume (ml)	99.68 ± 26.76
End systolic left ventricular volume (ml)	41.18 ± 17.37
Categorical variables	
Male (*N* (%))	66 (50.77)

*BMI*, body mass index; *BSA*, body surface area; *P*_*sys*_, systolic pressure; *P*_*dia*_, diastolic pressure; *EF*, ejection fraction; *PAPs*, pulmonary arterial pressure

### TAVI segmentation

ECG-gated CT images at 80% of the R-R interval corresponding to late diastole were employed for the segmentation of aortic root geometry using Mimics medical imaging software (v21, Materialize, Belgium). The segmentation process encompassed the isolation of the aortic root vessel, extending from the aortic valve annulus to the ascending aorta, as well as the identification of calcified plaques. Semi-automatic thresholding of contrast-enhanced images was employed, followed by manual editing and smoothing of the reconstructed mask, to obtain the aortic wall based on the connectivity of gray values within a dynamically selected range. A seed point is identified in the aortic lumen at the mid-ascending aorta, and the mask is then generated by comparing the gray value of the seed point with that of neighboring pixels. Segmentation automatically halts when the gray values deviate from the seed point by more than ± 70 Hounsfield units. Segmentation of the stenotic valve leaflets was omitted due to their thin structure not clearly visible at ECG-gated CT imaging. For the detection of calcification, a distinct mask was generated through fully automatic thresholding of bright plaque calcium [9]. The grey intensity value ranged from 1500 to 1850 Hounsfield units, with differences attributed to the ECG-gated CT imaging procedure.

The geometries of both the aortic root and calcifications were subsequently imported as stereolithographic files into Rhinoceros (v7, McNeel & Associates, USA) for further manual editing. Specifically, the aortic root surface was sectioned at a mid-height of the ascending aorta and just below the aortic root annulus for subsequent analyses. This approach is not based on an automatic cutting procedure, as described in other studies [10, 11].

### Statistical shape modeling implementation

An in silico virtual cohort of TAVI patients was developed using a SSM to generate the patient atlas, comprising the mean shape and its variations. The SSM was constructed within the MATLAB mathematical programming language (R2020, MathWorks Inc., Natick, MA, USA), as previously described in earlier studies conducted by our group on the aneurysmal aorta [12], left ventricle [13] and spine [14]. To generate a new virtual model, the mean shape of the TAVI patient population can be deformed based on desired standard deviation (σ) values for each shape mode. The latter allows capturing specific anatomical features of the TAVI patient population. The development of the in silico virtual cohort began with preprocessing segmented aortic root surfaces, followed by automatic alignment through registration and transformation algorithms and subsequent application of principal component analysis (PCA) for shape mode extraction.

After importing the aortic root and calcification surfaces into MATLAB, the geometries were resampled to sufficient resolution by reducing the total number of 3D point coordinates without altering the original vessel morphology, ensuring computational efficiency. Initially, random sampling of the original grid was performed to generate various refinement levels. Subsequently, the first shape mode was derived using PCA and plotted against the mesh resolution to assess the convergence of the surface grid. Convergence was indicated by a change of less than 5% in the first shape mode. This optimization process of geometric models resulted in 30,000 point coordinates for the aortic root wall and 50,000 points for the calcifications.

To develop the SSM, a reference patient model needs to be extracted from the patient population to align all aortic root and calcifications to the reference one. Alignment involved an initial rigid iterative closest point (ICP) transformation to ensure consistent orientation and position among shapes, followed by a nonrigid ICP for shape scaling to further enhance point cloud alignment. For both rigid and nonrigid transformations, the algorithm halted when the average difference between estimated rigid transformations in the three most recent consecutive iterations was less than the specified tolerance of 0.01 mm. A maximum of 120 iterations was set during which the function attempts to converge the two point clouds. The alignment process was distinctly performed for the aortic wall and calcification, as these shapes were common to all patients. For calcifications, alignment was performed subsequent to the alignment of the aortic sinuses (i.e., aortic wall and calcification) by manual rotation to ensure consistency from leaflet to leaflet. Then, alignment was carried out solely for the calcific plaques of each patient while relaxing the alignment tolerance parameter to 0.2 mm to avoid excessive rotation and translation. It should be noted that calcifications do not follow a predictable pattern across patients, and there is no consistent rule for how they correspond from one individual to another. Therefore, the approach used in this study assumes that calcific plaques are present on all valve leaflets across the entire patient population when determining the correspondence of calcification patterns.

Consistency in the final patient model was achieved as each aortic wall and calcification was aligned to the same reference shape. Given a potential bias in alignment with respect to the arbitrary reference shape, transformations were iteratively applied from the initial template shape to each rigidly aligned shape. This process was repeated using the mean shape surfaces as the reference shape. To further reduce bias, the preceding steps of rigid alignment, shape transformation, and subsequent rigid alignment were iteratively repeated until the average shape no longer changed. The aligned shapes were then prepared for PCA by concatenating the point coordinates of each shape into a vector, which were then assembled into a matrix comprising all patient models. PCA served as an unsupervised technique for extracting the required shape features essential for the virtual expansion of the patient cohort. The primary contributor to shape variability can be quantified by the first mode, with each subsequent mode capturing the highest remaining residual variance. The mean shape represents the average anatomy of the TAVI patient population, while the standard deviation measures the variability and deviations from this mean shape.

To evaluate the quality of the SSM, generalization was computed to assess the SSM’s ability to represent unseen data. This is done by calculating the average sum of squared errors using a leave-one-out procedure. In this approach, one patient is excluded each time, and a new statistical shape model is created using the remaining aortic roots. The new SSM is then used to reconstruct the shape of the excluded patient, and the difference between the original shape and the reconstruction is quantified by the mean squared error, progressively including additional shape mode. The generalization parameter is therefore given by:

1 $$GE=\frac{1}{N}\sum_{i=1}^{N}\Vert {x}_{i}-{\widehat{x}}_{i}(M)\Vert$$

where $N$ is the number of patients, ${x}_{i}$ and ${\widehat{x}}_{i}$ are the original and rebuilt left-out shape and $M$ is the shape modes.

### Virtual patient cohort generation

As a proof-of-concept, a cohort of 100 virtual patients was generated by randomly varying the statistical shape modes retaining the 90% of the overall shape variability upon a value of ± 2σ. This was achieved by varying the shape boundary in steps of 0.5 σ, both positively and negatively. The adopted deviation was decided after several attempts acting to deform the mean shape of the patient atlas without leading to unrealistic shapes or folded geometries. We also computed the deformed shape probability indicating the chance that a specific deformed shape can occur for a given value of the shape boundary (i.e., σ) to quantify the number of shape variations not included in our in silico cohort. To determine this probability, we used the Mahalanobis distance and the chi-square distribution. The Mahalanobis distance measures how far the deformed shape deviates from the mean shape in the context of the model’s variance. The squared Mahalanobis distance ${(D}_{M}^{2})$ follows a chi-square distribution assuming *k* = 3 degrees of freedom. The shape probability is given by the chi-square cumulative distribution function:

2 $$f\left(S\right)=\frac{1}{{(2\pi )}^{3/2}{\left|\Sigma \right|}^{1/2}}\text{exp}(-\frac{1}{2}{D}_{M}^{2})$$

where $\Sigma$ is the covariance matrix.

Validation of the synthetic patient cohort was assessed by comparing geometric parameters (i.e., the diameter of the aortic valve annulus and the volume of calcification) between synthetic and real aortic root models using both boxplot graphs and the Mann–Whitney *U*-test.

### Correlation and regression analyses

Pearson’s correlation was conducted to identify linear relationships of the shape modes with clinical and functional variables. To explore the association between shape features and disease status, a regression model was developed to predict the peak pressure gradient of the aortic valve stenosis (AS-PPG) using the shape modes resulting from PCA. The pressure gradient was obtained for each patient by Doppler echocardiography according to clinical guidelines. A subset of shape modes, retaining the six most influential shape modes based on their correlation with the target variable, was selected. A support vector machine (SVM) regression model was developed using Bayesian hyper-parameter tuning and tenfold cross-validation strategy to identify the optimal parameters while minimizing the root mean square error (RMSE) [15]. The *R*-squared values were used to explain the variance in the data captured by the model, but they do not indicate the predictive accuracy of the model. This analysis is relevant in clinical practice for developing a risk stratification strategy based on 3D anatomic geometry rather than local anatomical measurements.

### Machine learning for predicting optimal device size

The original patient population was categorized into two distinct groups based on the size of the implanted device, specifically those with the 23-mm device and those with the 26-mm device. This aimed to explore the predictive capability of the extracted shape features for determining the most suitable size of the S3 device for implantation. The shape features represented the principal component scores returned by the PCA analysis in MATLAB. For each patient, machine learning classifiers were adapted to predict the size-related group association. *F*-scores were calculated to select the principal component scores with the most significant impact on shape variations. Our predictive modeling approach involved four distinct machine learning classifiers: multilayer perceptron (MLP), logistic regression (LR), k-nearest neighbors (KNN), and SVM. Each model underwent training with the dataset divided into a training set (70%) and testing with the remaining data (30%), with the evaluation based on the area under the receiver operating characteristic (AUROC) curve. Confusion matrices were used to illustrate the performance of machine learning models, along with values of accuracy, recall and precision for each class.

## Results

**Fig. 1:**
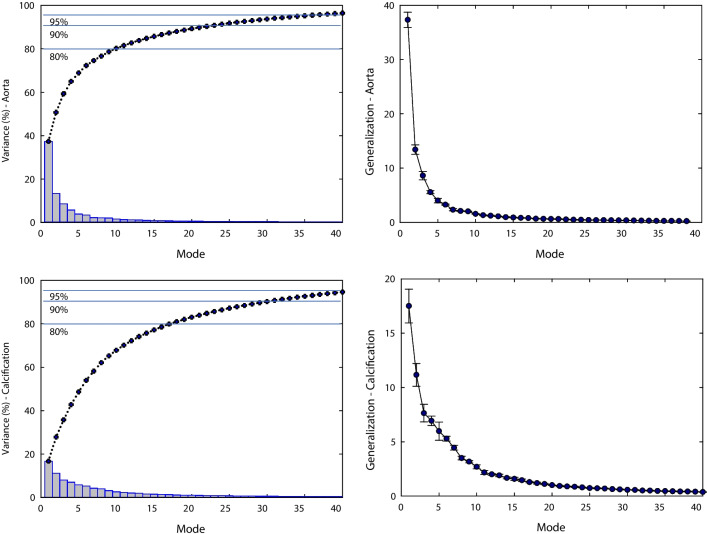
Profile of scree plot (left column) and generalization (right column) of both aortic root and calcification resulting from SSM

Figure 1 illustrates the scree plot, presenting the cumulative variance explained by each mode computed through PCA for the aortic root and calcification. To encompass 90% of the overall shape variability, the first 25 modes were necessary to account for the aortic root’s shape variability, while 32 modes were required for capturing the calcification variance.

**Fig. 2:**
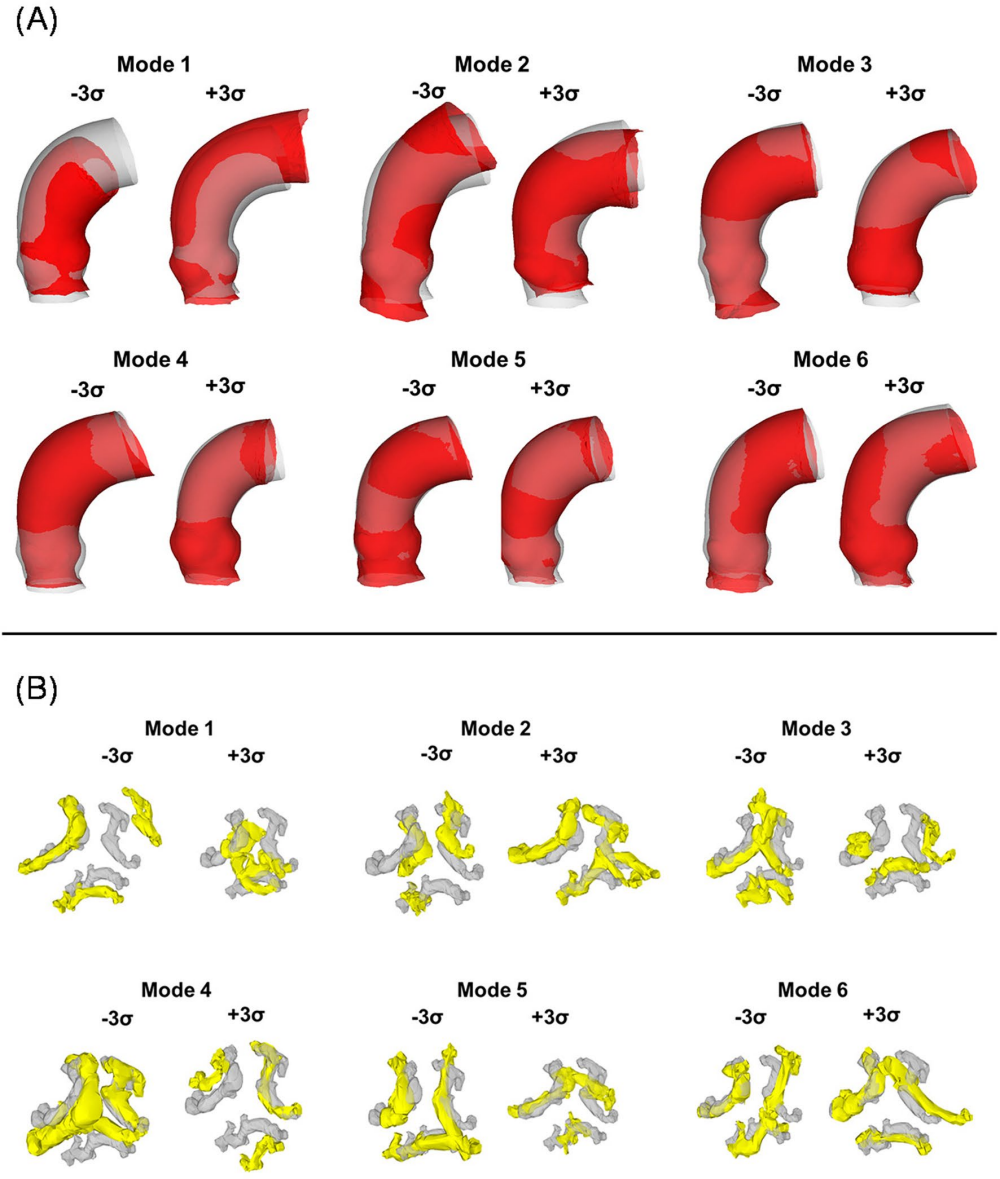
Dominant shape modes shown by deformations of the computed template from low (− 3 σ) to high (+ 3 σ) values for **A** aortic root and **B** calcification

The first six shape modes for both the aortic root and calcific plaques are depicted in Fig. 2. Mode 1 for the aortic root represents approximately 39% of the overall shape variability and correlates with proportional vessel size changes (scale factor). Mode 1 for calcification illustrates variations in the distances among calcific plaques, accounting for 19% of the shape variations. Mode 2 for the aortic root manifests changes in vessel curvature (10% of variance), while modes 3 and 4 correlate with aortic annulus (3.8%) and sinus (2.9%) dimension variations, respectively. Notably, mode 4 of the calcification variance is linked to changes in plaque volume (3.7% of variance), and mode 5 relates to plaque union among valve leaflets (2.7%).

**Fig. 3:**
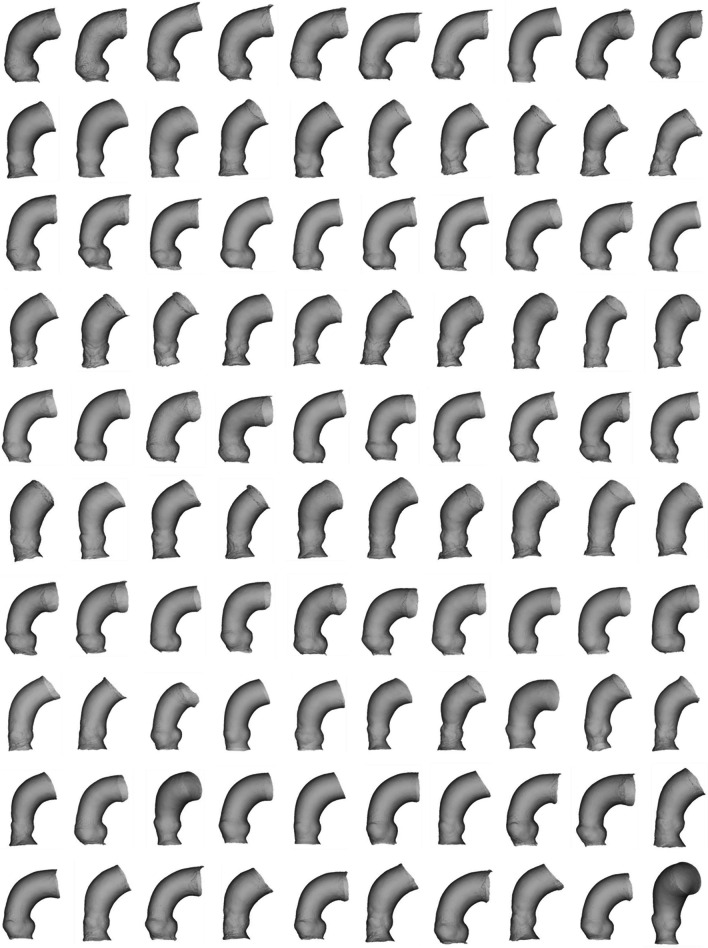
Synthetic data of n.100 virtual aortic root model

**Fig. 4:**
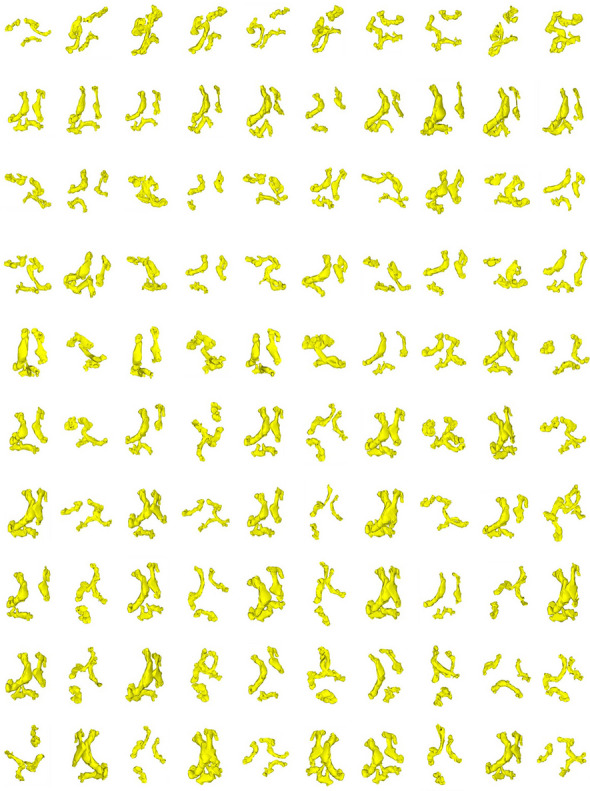
Synthetic data of n.100 virtual calcification

The in silico virtual cohort, expanded from the TAVI patient population, was effectively generated by deforming the first 25 shape modes of the SSM (Figs. 3 and 4). Qualitatively, none of the proposed anatomies exhibited overlapping or folded surfaces. Analysis showed a deformed shape probability of 30.85%, 15.87%, 6.68%, 2.28%, 0.62%, and 0.14% for shape deviations of 0.5σ, 1σ, 1.5σ, 2σ, 2.5σ, and 3σ, respectively. As the synthetic models were obtained within ± 2σ, this suggests that only a marginal proportion (i.e., < 2.28% of shape variations) of morphological variance was not considered in our in silico cohort.

**Fig. 5:**
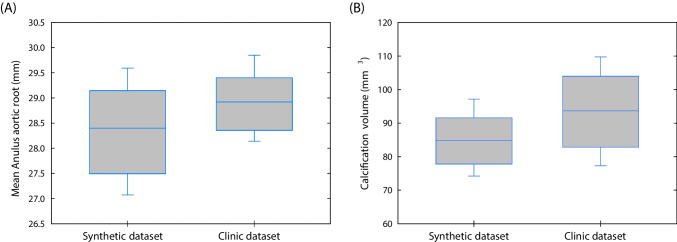
Boxplots comparing the **A** annulus diameter and **B** calcification volume between synthetic and clinical data

Figure 5 displays boxplot graphs comparing the geometry of synthetic models against the original patient dimensions. The median annulus size for synthetic models falls within the 50th percentile of that for TAVI patients, indicating that synthetic vessels generally mirrored true anatomy. However, the median values for synthetic calcification volume slightly exceed those of TAVI patients, suggesting that synthetic calcifications were marginally larger than actual calcific plaques on average. Not any statistically significant difference was observed at Mann–Whitney *U*-test comparison among groups (i.e., *p* = 0.425 for the aortic root and *p* = 0.318 for the calcifications). The discrepancies in the geometric measurement comparison can be attributed to several factors, including intra-operator variability and the lack of one-to-one correspondence.

**Fig. 6:**
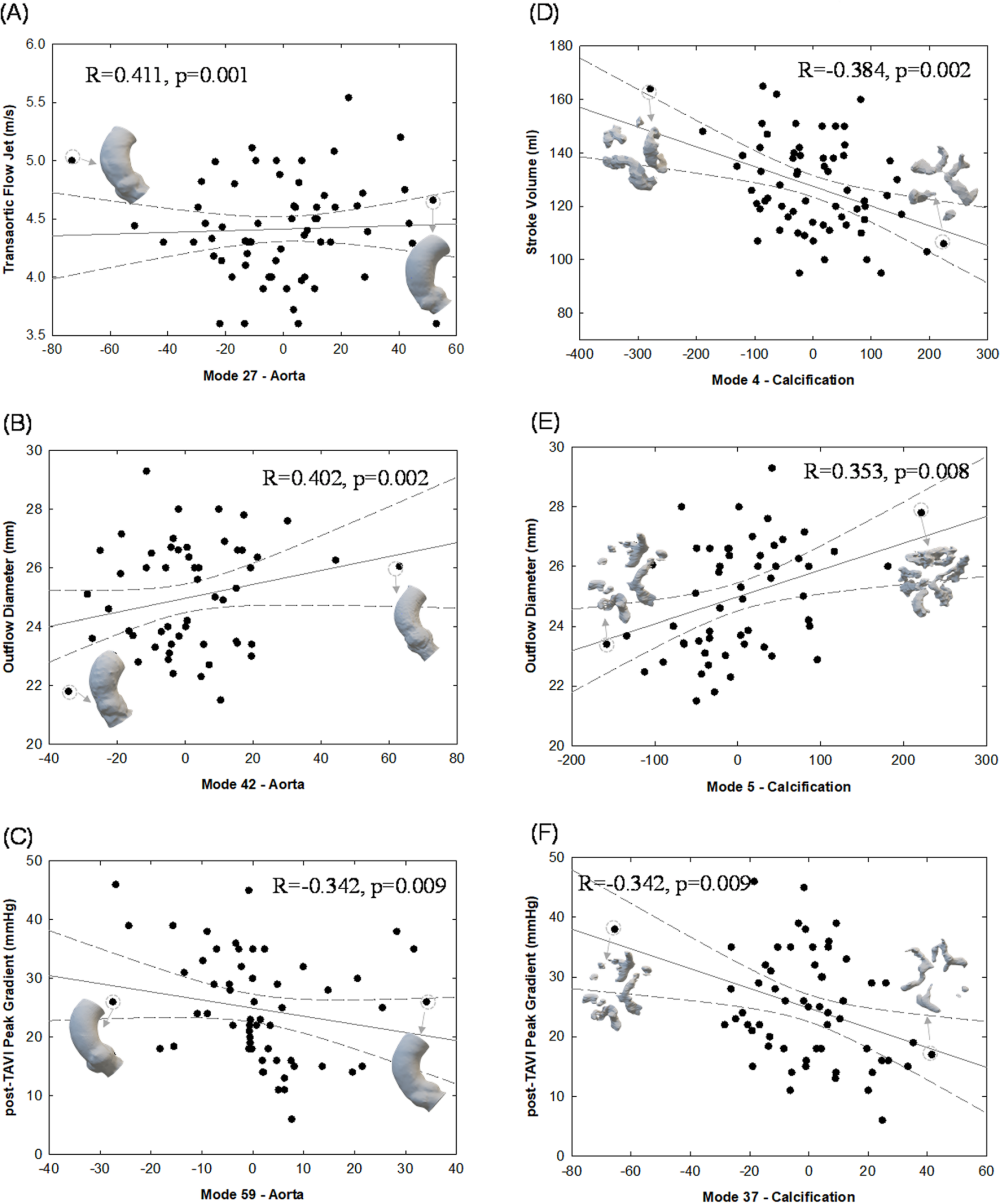
Pearson correlation showing associations of functional patient parameters with shape modes; (**A**) flow jet across the stenotic valve versus Mode 27 of the aorta; (**B**) device diameter at outflow versus Mode 42 of the aorta; (**C**) post-TAVI pressure gradient versus Mode 59; (**D**) patient stroke volume before TAVI versus Mode 4 of calcification; (**E**) device diameter at outflow versus Mode 5 of calcification; (**F**) post-TAVI pressure gradient versus Mode 37 of calcification

Figure 6 demonstrates various associations between shape features and functional patient data. A statistically significant positive relationship was observed between the transaortic flow jet and mode 27 of the aortic root (*R* = 0.411, *p* = 0.001), while mode 4 showed a negative association with left ventricular stroke volume (*R* =  − 0.384, *p* = 0.002). The peak pressure gradient across the implanted device was negatively correlated with both mode 59 of the aortic root (*R* =  − 0.342, *p* = 0.009) and mode 37 of the calcifications (*R* =  − 0.342, *p* = 0.009). The S3 diameter at device outflow exhibited a statistically significant positive relationship with both mode 42 of the aortic root (*R* = 0.402, *p* = 0.002) and mode 5 of the calcification (*R* = 0.353, *p* = 0.008).

**Fig. 7:**
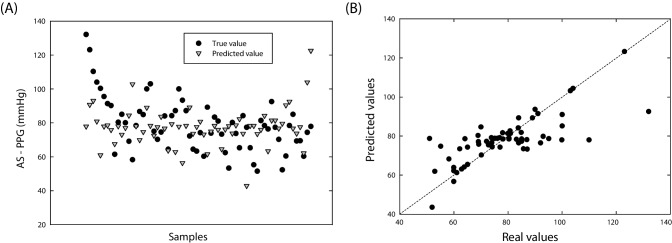
SVM regression of AS-PPG showing **A** the predicted and true response for each patient and **B** the comparison between predicted and true values

Regarding the regression of AS-PPG, Fig. 7 displays the comparison between real and predicted pressure gradient values for the SVM regression model. Utilizing the six shape modes, the SVM regression model with a polynomial kernel achieved an *R*-squared value of 0.551 and an RMSE of 11.67 mmHg after parameter optimization (best set with C of 11.079, degree of 3, and gamma of 0.149). Differences between actual and predicted values can be attributed to the predictive capability of the proposed regression model. A higher *R*-squared value indicates a smaller difference between actual values and predictions.

**Fig. 8:**
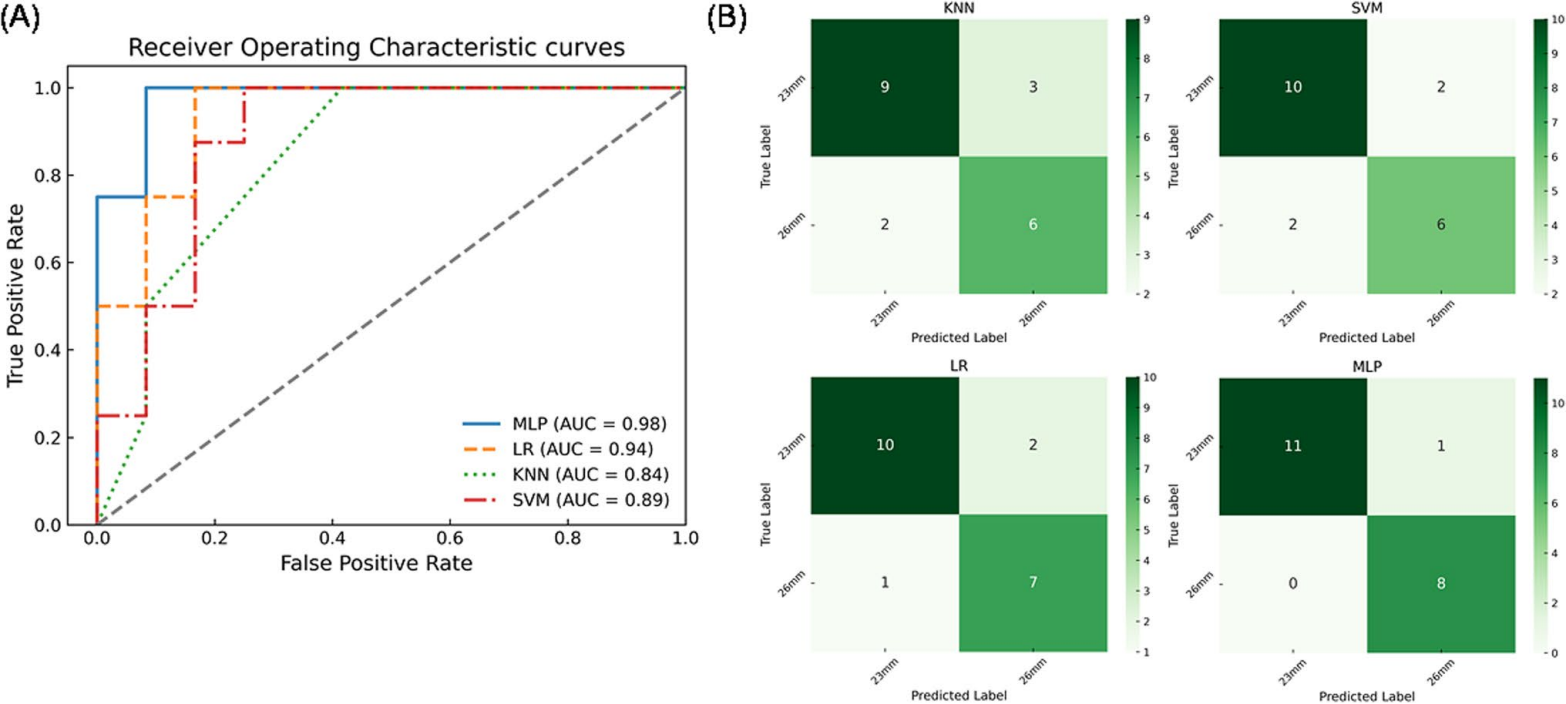
Machine learning classification showing **A** ROC curves and **B** confusion matrices for the investigated models

For classification, the multilayer perceptron emerged as the most effective model, exhibiting superior performance across evaluated metrics (refer to Table 2). Figure 8A showcases the ROC curves for the six shape mode classifiers. Among the machine learning models, the multilayer perceptron demonstrated exceptional predictive ability with very high sensitivity and specificity in determining the optimal device size for implantation using the six shape modes as classifiers (AUC = 0.98). A comprehensive assessment of model performance is depicted through confusion matrices in Fig. 8B.

**Table 2:** Classification scores obtained for machine learning models

	**MLP**	**LR**	**KNN**	**SVM**
Accuracy	0.95	0.851	0.750	0.800
Recall [23 mm]	1.00	0.909	0.818	0.833
Precision [23 m]	0.917	0.833	0.750	0.833
Recall [26 mm]	0.889	0.875	0.667	0.751
Precision [26 mm]	1.00	0.778	0.750	0.752
AUC-ROC	0.98	0.941	0.840	0.895

*MLP*, multilayer perceptron; *LR*, logistic regression; *KNN*, K-nearest neighbors; *SVM*, support vector machines

## Discussion

In this study, advanced statistical tools were utilized to assess the morphological variance of the aortic root in TAVI patients. This process facilitated the creation of a synthetic cohort of models designed for in silico trials, specifically aimed at evaluating the safety and effectiveness of transcatheter heart valves. This was achieved by developing a SSM using PCA to extract uncorrelated shape features from the original patient population treated with the S3 device. An ideal in silico cohort should not only reflect morphological shape variability but also encompass the physiological variance found within the original patient population. To address this, we first validated the synthetic models against clinical measurements and then assessed the association of extracted shape features with pre- and post-TAVI patient functionality. This investigation employed Pearson’s correlation and machine learning analysis, revealing that a subset of shape modes (i.e., principal component scores) could predict the severity of aortic valve stenosis by quantifying the pressure gradient via SVM regression. Moreover, our study demonstrated the capability of a multilayer perceptron machine learning model to predict the optimal choice of the intended device size using six shape-related classifiers. Such findings are relevant in the clinical setting, as the diameter of the aortic valve annulus alone may not suffice for assessing the optimal device size to be implanted. Here, we demonstrated that device sizing could be evaluated using 3D anatomic features, rather than relying solely on conventional 2D imaging or clinicians’ experience. This has the potential to enhance clinical decision-making processes. Overall, this study adds weight to the utilization of virtual cohorts in computational modeling and simulations. The synthetic data effectively captured the anatomical and physiological variability of TAVI patients, showing promise for diverse applications, including augmenting or optimizing clinical data sizes and investigating safety concerns associated with current or next-generation transcatheter heart valves.

### Virtual cohort for in silico trials

In computer modeling and simulations, conducting an in silico clinical trial enables the assessment of clinically relevant data regarding the performance of candidate biomedical devices through numerical simulations of the treatment’s physics [2, 16]. This approach not only reduces the costs associated with device experimentation but also enhances knowledge and confidence in device safety and patient outcomes. However, it is important to recognize that while in silico trials offer valuable insights, they come with inherent assumptions and constraints compared to in vivo trials. While the paradigm shift toward in silico trials is gaining traction, the methodology is still in its infancy in practical applications and regulatory sciences. In the context of TAVI, creating a virtual cohort for in silico trials necessitates capturing the variability of the patient anatomical population, including the degree of aortic valve stenosis and its functionality. Recent findings demonstrated the feasibility to treat degenerated transcatheter heart valves with a second transcatheter heart valve [17, 18], thereby expanding the applications of transcatheter heart valves to a new class of patients. It is therefore evident that the development and clinical assessment of TAVI device could benefit from in silico trials to overcome complications and drive knowledge in borderline anatomies and new patient classes [19]. To achieve this, SSMs employing PCA have proven to be a powerful technique for extracting shape variability, as highlighted by numerous studies in cardiovascular [20, 21] and musculoskeletal systems [14]. However, only a few studies have focused on developing and validating virtual cohorts using SSMs. For instance, Bridio et al. [22] generated a synthetic cohort of 100 cerebrovascular anatomies via random sampling of shape modes, ensuring the rejection of unphysiological anatomies based on defined acceptance criteria. La Mattina et al. [23] tested the femoral strength distribution in a virtual cohort generated with SSMs and compared it to a physical cohort, suggesting the feasibility of expanding virtual cohorts like to a phase III clinical trial. In the realm of TAVI, Verstraeten et al. [24] expanded a retrospective population of 97 stenotic aortic valves to a virtual cohort of 500 patients using non-parametric SSMs. However, their approach lacked investigation into calcifications.

In this study, we present a method that harnesses both local and global shape features of the calcific aortic root in TAVI patients and predicts the pressure gradient across the stenotic aortic valve using a set of shape modes. This approach aims to address the physiological variability of this population. As a proof-of-concept, this methodology allowed us to construct a synthetic cohort of 100 aortic root anatomies suitable for in silico TAVI trials, replicating realistic anatomic variability. Our results demonstrated that the geometric parameters of the generated virtual population aligned closely with those of the original patient population on average. Utilizing shape probability parameters, we showcased that the synthetic patient cohort spans a broad spectrum of anatomic variability, with less than 3% of shape boundaries excluded during virtual model generation. This ability to capture a wide range of variations is crucial, particularly for worst-case scenario considerations if the proposed SSM approach is utilized for expanding the original patient population. Moreover, correlation and regression analyses were conducted to quantify the associations between shape and function. These statistical approaches allow for the extrapolation of aortic valve function estimates, such as the pressure gradient across the stenotic aortic valve, for each synthetic model. Therefore, while the anatomical accuracy of our synthetic cohort is well-established, its functional capability is less confirmed. For a comprehensive analysis of in silico trial requirements and development, the work by Bischoff and collaborators [16] is highly recommended.

### Assessment of shape features

In the field of TAVI, the study by Bosmans et al. [25] stand as the sole demonstration of the utility of a PCA-based SSM in determining the optimal size of transcatheter heart valves using 3D shape features. Their findings highlighted the necessity of the first twenty shape modes to capture 95% of the overall shape variability. Specifically, they associated the first shape mode with general size variation and the second with vessel curvature and angle with respect to the aortic annulus. These findings align well with our SSM, confirming the compactness and shape boundaries observed in the aortic root. Moreover, our SSM demonstrated robustness in representing unseen patient data, as indicated by the generalization parameter. Notably, our study is unique in exploring variability within calcific plaques, identifying specific shape features associated with clinically relevant parameters such as calcification volume (e.g., mode 4).

An alternative method to PCA for SSM development involves partial least square analysis, enabling the creation of a clinically oriented statistical shape decomposition. Bruse et al. [20] applied partial least square analysis to develop an aortic coarctation SSM based on dependent variables like ejection fraction and body surface area. They observed statistically relevant correlations between functional-derived shape modes and clinical measurements. Recently, Geronzi et al. [26] employed both PCA and partial least square analysis to implement an SSM of the aneurysmal ascending aorta and predict dilatation rates. While lacking comparative analysis, they noticed a slight difference in the local shape variation extraction capabilities among the two decomposition techniques. Specifically, both the first principal component and the partial least square shape mode correlated with the pattern of aortic dilatation. However, PCA was linked to overall vessel size, while partial least square analysis was associated with aneurysm diameter. Nevertheless, the overall compactness and generalization were comparable between the two decomposition techniques.

### Correlation and machine learning

Several associations between shape modes and patient function before and after TAVI were observed, shedding light on hemodynamic and structural mechanics. Notably, the correlation between mode 27 of the aortic root and the transaortic flow jet of the stenotic valve suggests that sinus shape influences blood flow dynamics, potentially contributing to aortic stenosis development in our patient population. Similarly, the link between high calcification volumes (represented by changes in mode 4) and low stroke volumes aligns with common findings in severe aortic stenosis patients [27]. Moreover, our observations indicate that a combination of plaque union among leaflets (mode 5) and the circumferential dimension of the aortic root (mode 42) can estimate the device size at implantation. Additionally, high transmural pressure across the implanted device is associated with aortic roots having small annulus dimensions (mode 59) and large calcifications (mode 37).

In regression analysis, the *R*-squared value of 0.551 indicates that the SVM regression model can explain approximately 55.1% of the variance in the pressure gradient of the original patient population, with a model deviation of about 11.67 units from actual values. While this suggests that the SVM model captured a significant portion of pressure gradient variation based on selected shape modes, there might be room for further enhancement. Exploring different model architectures, feature engineering, or alternative machine learning algorithms could potentially improve predictive performance. In similar studies, SSM were combined with computational fluid dynamics to predict flow across the aortic valve by meta-models [28] or deep leaning [29].

Combining computational predictions with machine learning techniques, Galli et al. [30] showcased the predictive capability of machine learning classifiers in estimating the probability of developing TAVI-related conduction abnormalities. However, to the best or our knowledge, this is the first study proposing machine learning classification using SSM-derived shape features rather than clinical variables. Here the MLP model—a neural network adept at deciphering intricate patterns—demonstrated remarkable robustness and effectiveness in handling the classification task, achieving high accuracy, recall, precision, and a high AUC-ROC. With an AUC-ROC of 0.98, the MLP model demonstrates a strong ability to differentiate between different sizes of the S3 device based on shape features. Moreover, the model achieved approximately 91.7% accuracy when predicting the 23-mm device size and around 88.9% accuracy for the 26-mm device. Further analysis might involve examining misclassifications and exploring methods to maintain or enhance these scores on unseen data. Although the in silico cohort was developed before TAVI, the predictive capability of the machine learning model presented here is specific to the S3 device and cannot be extended to other transcatheter heart valves.

### Study limitation

The main limitation of the proposed statistical shape atlas lies in its inability to represent the native valve leaflets. CT imaging inadequately captures the thin structure of these leaflets, making their reconstruction challenging using thresholding techniques. Various research groups have explored parametric modeling based on anatomical landmarks to address this limitation [31, 32]. However, due to uncertainties in native leaflet shape and reconstruction, the statistical shape analysis and resultant synthetic cohort lack representation of native valve leaflet variability. A potential solution involves initially generating the synthetic aortic root model and subsequently reconstructing valve leaflets using parametric surfaces and imaging measurements. Moreover, the approach to aligning calcifications began with an initial alignment of the aortic sinuses. This method may result in inconsistencies with each patient’s calcification patterns. Calcifications can occur at various locations, posing challenges for alignment and point correspondence. The approach used to develop the SSM and establish correspondence between patients assumes calcification on all three leaflets in every patient, limiting its reliability. A conditional probabilistic model describing the distribution of calcifications based on their location on the leaflets could provide a more suitable alternative in this scenario.

The second limitation pertains to the validation method, which primarily compared annulus dimensions between synthetic and real patient models. It is essential to acknowledge that the SSM inherently relies on the dataset used for its construction. Therefore, to ensure robustness, synthetic data should exhibit parameter distributions comparable to those of an independent dataset. A better strategy would be to construct a SSM from a subset of the original clinical cohort, utilize the virtual population for training purposes, and subsequently evaluate the model on the original clinical subset that was not included in the SSM. Finally, while machine learning analysis showed high predictive capability, validating the model on a separate test dataset, or using a large patient cohort can provide an improved assessment of the predictive model capability. The study included a TAVI population as homogeneous as possible, excluding patients with bicuspid stenotic aortic valves. As a result, the in silico cohort did not encompass the full spectrum of the TAVI patient population.

## Conclusions

The study advanced our understanding of the association between vessel shape features and clinical outcomes among TAVI patients, leveraging statistical shape analysis and machine learning. Particularly noteworthy was the successful development of synthetic data, enabling emulation of anatomical and physiological variability. This achievement holds significant promise in augmenting in silico trials, where cardiovascular devices are tested on virtual patient groups represented by computer models. Additionally, our findings suggest the effectiveness of machine learning in accurately predicting disease status and distinguishing between various sizes of TAVI devices based on patient morphological features.

## Data Availability

The datasets generated during and/or analyzed during the current study are not publicly available due to ethical issues but are available from the corresponding author on reasonable request.
